# Supplementary material for: A genetic toolkit for tagging intronic MiMIC containing genes
Source: eLife. 2015 Jun 23;4:e08469. doi: 10.7554/eLife.08469 (PMC4499919; doi:10.7554/eLife.08469)
Supplement: Figure 1—source data 1. — DOI: http://dx.doi.org/10.7554/eLife.08469.003 [file elife08469s001.doc]

**Figure 1 supplemental data 1:** List of constructs

| **Construct** | **Description** |
| --- | --- |
| pW35-FRT-attB-SA-phase0-(GGS)_4_-EGFP-FlAsH-StrepII-TEVcs-3xFlag-(GGS)_4_-SD-attB-w^+^-FRT | Splice phase 0 EGFP-FlAsH-StrepII-TEVcs-3xFlag plasmid |
| pW35-FRT-attB-SA-phase1-(GGS)_4_-EGFP-FlAsH-StrepII-TEVcs-3xFlag-(GGS)_4_-SD-attB-w^+^-FRT | Splice phase 1 EGFP-FlAsH-StrepII-TEVcs-3xFlag plasmid |
| pW35-FRT-attB-SA-phase2-(GGS)_4_-EGFP-FlAsH-StrepII-TEVcs-3xFlag-(GGS)_4_-SD-attB-w^+^-FRT | Splice phase 2 EGFP-FlAsH-StrepII-TEVcs-3xFlag plasmid |
